# Meeting the reproductive health needs of female sex workers in Côte d’Ivoire: protecting the human right to dignified health

**DOI:** 10.1186/s12978-023-01659-z

**Published:** 2023-09-05

**Authors:** Nika Elmi, Nuria Gallego Marquez, Katherine Rucinski, Carrie Lyons, Gnilane Turpin, Ibrahima Ba, Nguissali Turpin, Emile Gouane, Evelyne Obodou, Daouda Diouf, Stefan Baral

**Affiliations:** 1 Center for Public Health and Human Rights, Department of Epidemiology, Johns Hopkins School of Public Health, Baltimore, MD USA; 2 ENDA Santé, Dakar, Senegal; 3 ENDA Santé Côte d’Ivoire, Abidjan, Côte d’Ivoire; 4 Department of International Health, Johns Hopkins School of Public Health, Baltimore, MD USA

**Keywords:** HIV, Reproductive health, Unintended pregnancy, Sex work, STI, Contraception

## Abstract

The sexual and reproductive health needs of female sex workers (FSW) are often understudied and underserved in the context of HIV-related research in countries across Sub-Saharan Africa and West Africa. We assessed the lived experiences of FSW across Côte d’Ivoire to characterize unmet reproductive health needs and opportunities to address them. From February-August, 2020, ENDA Santé, Côte d’Ivoire conducted 75 in-depth interviews and 15 focus group discussions with FSW and community informants in five cities in Côte d'Ivoire. Themes that emerged included the inconsistent use of contraception services, a history of unintended pregnancies, and experiences of stigma at public healthcare facilities. Opportunities to increase the impact of both SRH and HIV services included strengthening existing HIV and family planning service integration for FSW. Taken together, the results highlight the importance of addressing the unmet reproductive health needs of FSW to both optimize the HIV response and increase the delivery of human-rights affirming sexual and reproductive health services for sex workers.

## Plain language summary

In Côte d’Ivoire, female sex workers (FSW) continue to have an unmet need for sexual and reproductive health (SRH) and HIV prevention services. The disproportionate burden of HIV/STIs is driven by several shared factors including behavioral, social, and structural determinants of HIV, STIs, and pregnancy; as well as biological efficiency of transmission of HIV in the context of STIs. This qualitative study examined the unmet SRH needs of FSW in 5 cities in Côte d’Ivoire to better understand how to offer integrated and high quality SRH and HIV programs and services. Working together with ENDA Santé Côte d’Ivoire, an organization that works closely with FSW, 75 in-depth interviews were held among FSW and community informants, and 15 focus group discussions with FSW. After analyzing the transcripts, several key themes emerged including use of contraceptive methods and unintended pregnancy, the experience of pregnancy and childbirth, HIV care access and quality, FSW-differentiated healthcare services and providers, and opportunities for service integration. The findings from this study underline gaps in the health system for FSW and the benefits of including SRH programming into routine HIV services to strengthen existing efforts.

## Background

In countries across West Africa, an estimated 19 million women of reproductive age have an unmet sexual and reproductive health (SRH) need for contraception, and 33 million women want to avoid a pregnancy [2]. The burden of HIV remains high with an estimated five million adults and children living with HIV in 2021 [3]. SRH and HIV are linked by several factors including shared behavioral, social, and structural determinants of HIV, STIs, and pregnancy; as well as biological efficiency of transmission of HIV in the context of STIs. The provision of dignified SRH coverage requires appropriate consideration of HIV epidemiology and overlapping root causes including poverty, discrimination, and gender inequality [4].

HIV continues to have a disproportionate impact on female sex workers (FSW) around the world. FSW have a 30-times greater risk of acquiring HIV compared to other adult women [5]. The occupational risks associated with sex work magnify the risk of HIV transmission and other STIs [6]. The burden of STIs has been estimated to be about 15 times higher among FSW than the broader population of reproductive aged women [7–9]. In Côte d’Ivoire, the unmet HIV prevention needs of FSW were estimated to account for 95% of all new HIV infections during the early years of the HIV epidemic between 1976 and 1985, declining to an estimated one in five new infections from 2005 to 2015 [10]. Although HIV prevention campaigns that focus on supporting FSW have been implemented regionally since 1991 [11], FSW remain particularly vulnerable to HIV infection due to multiple intersecting stigmas and suboptimal programming that has not sufficiently met the SRH and HIV needs of FSW [12].

In Côte d’Ivoire, low contraception use, barriers to care, and a high burden of unintended pregnancy have been drivers of poorer reproductive health outcomes among FSW [13]. Maternal mortality rates in Cote d’Ivoire were estimated to remain high at 473–604 deaths per 100,000 live births between 2000 and 2020 [14, 15]. A significant contributor to the high rates of maternal morbidity and mortality are unsafe abortions caused by legal restrictions prohibiting pregnancy termination unless the life of the woman is threatened [16]. The National Strategic Plan of 2016–2020 in Côte d’Ivoire prioritizes FSW as a key population that is disproportionately impacted by HIV due to varying levels of HIV knowledge, stigma, inconsistent condom usage, challenges in condom negotiation, and exposure to sexual violence [17]. However, barriers still exist to reaching and accessing appropriate healthcare due to fear of discrimination from within the community and in health care facilities [18, 19]. These structural limits hinder already marginalized sex worker communities from safely actualizing their reproductive health goals, including deciding on the number and timing of pregnancies.

There has been consistent evidence highlighting the need to better integrate SRH and HIV services as an important strategy for achieving HIV epidemic control. Poor SRH outcomes and increased exposure to HIV emerge from overlapping factors in sex work including economic instability, gender inequality, stigma, and sexual networks [20]. The need to address the bi-directional linkages between SRH and HIV programming is a concept that is widely recognized in the literature [21–23]. A comprehensive approach to HIV has been discussed for nearly 20 years, beginning with the 2004 Glion Call to Action’s recommendations to link access to reproductive health services with HIV prevention [24]. In 2005, the World Health Organization highlighted four priority areas for HIV/SRH linkage including learning one’s HIV status, promoting safer sex, optimizing the connection between HIV and STI services, and integrating HIV with maternal and infant health [25]. The Joint United Nations Programme on HIV/AIDS (UNAIDs) 2025 targets also integrates SRH as part of addressing the HIV epidemic [26]. Despite early endorsement of this approach, FSW were not specifically mentioned in these movements, and many barriers still exist to effective in-country scale-up and programming [27, 28]. The world is not on track to achieving Sustainable Development Goal 3.3, achieving the end the HIV pandemic by 2030, due to barriers including stigma associated with HIV and sex work, and a lack of synergy between FSW-specific HIV and SRH programming [29].

There remains limited evidence to guide large programs in Côte d’Ivoire and to scale-up integration efforts among FSW. In response, this study explores the unmet SRH needs of FSW in Côte d’Ivoire to better understand how to offer SRH and HIV programs and services so as to uphold the standard of health as a human right.

## Methods

### Study setting and data collection

Qualitative data were collected as part of a mixed methods bio-behavioral survey and programmatic study involving quantitative and qualitative data collection across five cities in Cote d’Ivoire: Aboisso, Soubré, Agboville, Yamoussoukro, and Katiola. Data were collected between February and August 2020 by ENDA Santé Côte d’Ivoire, an organization that works closely with FSW and other highly vulnerable key populations. The 5 cities were selected by ENDA Santé due to their large networks of FSW and NGOs for FSW. 1177 participants were enrolled in the quantitative study. The qualitative component of the parent study included focus groups and individual interviews with FSW and key informants (KIs) in the community who has knowledge about the sites frequented by FSW including cab drivers, police officers, or bar staff. In total, 75 in-depth interviews (IDIs) were held among FSWs (n = 50), KIs (n = 25), and 15 focus group discussions (FGDs) with FSW. Participants were compensated 5000 FCFA (approximately $8.17 USD) for their participation in individual interviews or focus groups.

### Focus group discussions and in-depth interviews with FSW

Respondent driven sampling (RDS) was used to recruit FSW for the quantitative arm of the study. Seeds for RDS were collected from diverse backgrounds and criteria to maximize the possibility of recruiting FSW of diverse ages, levels of education, marital status, practices of sex work (i.e. FSW working in brothels or those that work discretely), and HIV status. Inclusion criteria included being 16 years or older, having lived in the study area for at least three months, and having provided informed consent to participate in the study. Informed consent could not be provided if the participant was under the influence of substances or had any condition that impeded on their ability to understand the study, questions, or procedures. Participants were invited to participate in the qualitative study following the completion of the quantitative component. For the qualitative component, a convenience sample was selected among participants in the quantitative study to represent a diverse background including FSW that had experienced discrimination, stigmatization, drug usage, and had varying understandings of prevention practices for STIs/HIV. Training was provided by ENDA Santé to all research members as part of the overall study to explore how to structure interviews, how to ask questions and probe responses, and how to request and receive informed consent.

The FGDs among FSW probed the following topics: sociodemographic characteristics, initiation into sex work, condom use, barriers and facilitators to accessing HIV care, access to reproductive health services, suggestions for improving prevention and care services for FSW, the location of the health services used, and stigma (anticipated or experienced). On average, 2–3 FGDs were conducted per locality with 6–8 participants in each discussion. 12–24 FSW were included per locality, and 10 FSW of different profiles, such as individuals with exposure to violence or substance use, were selected through convenience sampling for individual in-depth interviews. Individual interviews with sex workers also involved conversations surrounding the experience and practice of sex work, knowledge and practice of HIV prevention, and suggestions for improving prevention and care services in place of sex workers. The same FSW were not selected for both FGDs and IDIs.

### In-depth interviews with key informants

A total of 25 interviews were conducted with community stakeholders recruited through convenience sampling including NGO staff, healthcare workers, police officers, bar workers, and taxi drivers. A semi-structured interview guide was used to explore the experiences of FSW in Côte d’Ivoire. IDIs with key informants included service offerings and structural barriers to health care, community dynamics of sex workers, knowledge about HIV and HIV risk behaviors, and mental health and drug and alcohol use among FSW. Interviewees as well as key informants were recruited by study staff.

Inclusion criteria for community informants included being 18 years or older and having knowledge about sites frequented by FSW to meet partners. Participants were not included in the study if they were under the influence of substances or unable to provide informed consent.

### Ethics statement

All data were collected in accordance with established national ethical guidelines set by the Côte d’Ivoire National Research Ethics Committee. The Johns Hopkins Institutional Review Board also approved the secondary data analysis presented here. The study was completely anonymous with de-identified codes used at all stages to track participants. The names of the FSW or the organizations they worked for were not collected or associated with interview transcripts. To guarantee confidentiality and respect for human dignity, best practices for collecting informed consent and data protection were put in place [30].

### Data analysis

All interviews were recorded and transcribed for analysis in French—the official language of Côte d'Ivoire. The quotes below were translated to English. The texts were coded and analyzed using NVivo software on inductive and deductive themes [31]. Identifying unmet reproductive health needs guided the creation of a codebook prior to beginning thematic analysis. Following this, one investigator reviewed the transcripts multiple times to refine the codebook. Relevant data were highlighted and turned into the production of initial codes and themes. The data were reviewed again using this codebook to generate final themes by reviewing the codes across the data set. The Structural Determinants HIV Framework was adapted using analytic memos and thematic analysis [32]. This framework displays the identified themes in a way to organize and highlight the relationships between themes. Data saturation was reached when no new themes or concepts emerged from the data.

## Results

Six themes emerged from this analysis including: inconsistent use of contraceptive methods, unintended pregnancy, the experience of pregnancy and childbirth, HIV care access and quality, FSW-differentiated healthcare services and providers, and opportunities for improved service integration.

### Inconsistent use of contraceptive methods

Some FSW displayed an awareness of common family planning methods including contraception such as implants and oral contraception pills, while others discussed traditional methods including the “rope and small balls method”:

*They do family planning. Others take the pill, there are others who have implants. There’s traditional too. It’s a rope that you put on and as long as the rope is not cut, you can’t getpregnant. There are also small balls, if you want five (5) years without having children, you swallow 5 balls. They do this in Katiola, Képlélé, Katinon, Korhogo and Dabakala as well. [KI, IDI, Katiola]*

*For condoms also when you come, they [health services] are given to you whether you are sick or not so as not to contaminate others and not to have illnesses, pregnancies, HIV yourself, just to protect yourself with others. We can also be given pills, IUDs, implants, there are many prevention methods. I forgot too. [FSW, IDI, Soubré]*

Some women utilized contraception methods not recommended by the Côte d’Ivoire Ministry of Health, World Health Organization (WHO) or Centers for Disease Control and Prevention (CDC) such as drinking lemon or purging the body using cotton leaves:

*I drink vinegar to destroy the sperm so that I don’t get pregnant when I finish. Some of my peers say they drink lemon or they purge with the cotton leaves. The others, I’ve never tried them, but vinegar—a lot of girls who do the work know about. [FSW, FGD, Yamoussoukro]*

There was widespread understanding among FSW participants that condom use was important and an impactful way of preventing HIV transmission and unintended pregnancy. Among some FSW, as familiarity with the client increased over time, the likelihood of condom use decreased. In most cases, FSW demanded higher prices for clients who preferred to forgo condom use. The financial influence underlying the absence of barrier contraception could be a motivator for FSW due to the financial pressures and obligations.

*I use condoms most with occasional clients because I don’t know them. But with regulars aren’t all the time. Clients I don’t use condoms with give me a lot of money. When, that’s the way they are, they say they pay a lot of money so they don’t have to wear so, we do like this. [FSW, IDI, Aboisso]*

Participants also discussed experiences of condoms breaking and agreeing to continue without a condom for increased pay. A participant under 18 discussed strategies clients use to proceed without contraception:

*I have protected sex with clients, but if during sex the condom broke there, they’re already in! It’s already in, “if you have it there, I'll take it!” So there, you increase [pay] and then you do without! [FSW, FGD, Katiola]*

There was agreement that condoms could be used to avoid STIs including HIV, and to prevent unintended pregnancies:

*The advantages, you can’t have STIs, you preserve yourself you can’t get pregnant. Everything that is sick you can’t have, you are comfortable in body, you can have malaria but diseases that contaminate from men there you can’t have. That’s why you have to protect yourself. [FSW, Focus Group, Aboisso]*

Younger FSW expressed limited negotiating power when it came to condom use. These participants were more likely to accept higher pay from clients who refused to wear condoms, and were more vulnerable to intimidation, violence, or other negative influences:

*Also the gain is a little easier because if the client offers them a little more, she will think that she will earn a little more, so they are a little more exposed, not to mention the fact that when you are young, you are still immature, and when they start this activity, tears and wounds are frequent and they leave entry points, so I think that age is a risk factor [KI, IDI, Agboville]*

### Unintended pregnancy

Findings from both FGDs and key informant interviews found that FSW who experienced an unplanned pregnancy reported a desire to have an abortion. Reasons for abortion, referred to below as ‘kidnap’, included lack of support from the involved partner and insufficient resources to raise the child:

*Often when they terminate there, it’s because the one who knocked her up refuses to take responsibility. They ask themselves, ‘When I give birth, how am I going to feed him? How am I going to take care of him?’ That's why they prefer to kidnap to get on with their lives. [FSW, FGD, Katiola]*

Traditional healers and medicine were used to terminate pregnancies among some FSW:

*When they want to remove the pregnancy, it is traditional they do or Chinese medicines. In the market there are shacks where Chinese medicines are sold for 15,000 francs. The treatment lasts three days. You take it every night. But you don’t have sex and you mustn’t eat sweet things during the three days; the third day it goes down. I’ve done that once. I know co-workers who have done it a few times. [FSW, IDI, Katiola]*

The leaves of the cotton plant were used as a traditional method to clear the body, or “purge”, to terminate and avoid pregnancy:

*Yes, there’s the new leaves of the cotton plant coming out, you take that to purge yourself. It’s very effective. [FSW, IDI, Katiola]*

### Experience of pregnancy and childbirth

Regarding pregnancy among FSW, some chose to continue working until childbirth while others remained at home and stopped seeing clients.

*They don’t have time, even during pregnancy they are doing the job. A day like this, it can take it and then they will send it to the hospital. And then she gives birth. Me when I am pregnant, I am quiet because that I follow the appointments until delivery. I don’t even go out, I stay at home until I give birth [FSW, IDI, Katiola]*

Some participants use health centers for childbirth, but some rely on other FSW for birth support.

*FSW is not written on our foreheads. We give birth in hospitals, in maternities, in clinics. I give birth at the clinic. But, some return with family, some also remain in the “ghettos”. We have sisters who are at “Ghetto” with their babies. Well, we are mothers, we “manage” [KI, IDI, Yamoussoukro]*

### HIV care access and quality

FSW reported that HIV prevention services are generally available and functional, both in hospitals and with NGOs in their communities. FSW expressed greater ease at NGOs because of the providers’ greater understanding and compassion toward sex work:

*We need to set up screening, counseling and prevention corners for FSW like DIC [drop-in centers] there. There they will feel they’re comfortable. [KI, IDI, Soubré]*

While prevention services were generally accessible, many FSW reported experiences of discrimination due to enacted stigma at hospital sites. FSW generally chose not to disclose their sex worker status in fear of experiencing enacted stigma or discrimination. Experiences of enacted stigma were commonly reported at health centers:

*Most of the public facilities, when they see that you have a problem with sex, they start calling you names: you’re selling yourself…stuff like that though, they’re there to treat us. It’s to humiliate the people there as if they are not there looking for the money. When it’s like that, it’s frustrating. [FSW, FGD, Agboville]*

Participants noted that counselling and prevention services could be improved by allocating more time to FSW education about ARTs and their health impact. Improving health literacy surrounding HIV treatment is seen as an effective approach to increasing HIV treatment uptake. Comprehensive and compassionate HIV counselling was indicated as an effective way to empower the community with knowledge on HIV prevention and treatment.

*We need to improve the counselling service. When it comes to taking medication, doctors need to take the time to explain to patients because some patients complain that the medication makes them tired. Some patients complain that the medication is tiring, others take it today, but tomorrow they don’t. If you advise the person properly, they know that if they don’t take the medication properly, they may die. [KI, IDI, Aboisso]*

There was also an awareness of pre-exposure prophylaxis (PrEP) in the community as a way to protect against HIV. Some FSW were also knowledgeable about services in their area that offered treatment.

*There is now PrEP, there is continuous PrEP and there is discontinuous PrEP. I take the continuous one. Often because when you know that you have a "brothel to manage" and then you know that your appointment will be without a condom you report that and you are given 4 tablets. You take the day before 2, 1 the day of, and then the next day. Even if the person is infected, you are protected. It is this method that we often use, otherwise it is the condom. [KI, IDI, Yamoussoukro]*

*I can say that they are very well organized. When you go they explain everything you need to know. Me, really, it's there that I like. They explained to me that I am at 7% so I am taking my medication well. The day they told me I thought it was over for me. But, it is the mother who follows me who assured me that life goes on that taking my medication in any case, it was well organized. [FSW, IDI, Soubré]*

### FSW-differentiated healthcare services and providers

Many FSW reported that healthcare workers should be empathetic, have an understanding of sex work as an occupation, and should prioritize confidentiality. Many safety and security concerns were associated with breaches in confidentiality that exposed a participant’s serological status. FSW noted experiences of their sex worker status or their health status, being made known publicly. Health workers who prioritized confidentiality and understood the safety risks and concerns of the FSW were essential to trusted and accessible HIV care:

*The way they are received in the first place. Secondly, to talk to them. Thirdly, it is the fact of feeling in confidence and then to tell them that this place is open to you and that if you have a small problem don’t think that we will judge you or stigmatize you. This place is like a home, we are a family. When a FSW hears that she feels safer here than at home or with her friends. The way of receiving them, the confidentiality. [FSW, IDI, Soubré]*

Participants agreed that services specific for FSW care would be more approachable as they address barriers to care such as stigma.

*I can say is to put an emphasis on awareness and then also create other NGOs for us sex workers. If there are more NGOs for us, in any case we will be good too because we cannot all gather at the hospital there because we will always feel a little embarrassed; so if there are more NGOs it is good and we must emphasize even awareness [FSW, FGD, Agboville]*

### Opportunities for service integration

Many FSW highlighted the importance of combining services such as integrating condom distribution together with HIV testing. Counselling was highlighted as an important factor to HIV care and service provision.

*What is important for me is the testing, which is important for us with the distribution of condoms, that is also important for me, so that's all I remember, we have to increase it. [FSW, IDI, Agboville]*

*You have to counsel well first because if there is no counseling there if she wants there if you want to there she will always catch disease there so you have to counsel her well. [FSW, IDI, Agboville]*

Participants indicated the importance of a comprehensive and multi-faceted approach to HIV prevention. A holistic framework is favored to promote overall health through awareness, counselling, and screening.

*As we say, everything is done in stages, you can’t skip one stage and go into another. There is the prevention service, the screening service, that is to say, if you have not been sensitized, you cannot be screened, that is to say, sensitization is important. So we can’t skip one step and fall into another. All the services are important and now everything is working well. [FSW, FGD, Agboville]*

## Discussion

In Côte d’Ivoire, it is widely recognized that FSW face many unmet needs regarding SRH beyond HIV prevention and treatment, including exposure to STIs and unintended pregnancies—gaps that contribute to increased mortality and morbidity among FSW [33]. While there have been critical advances in SRH and HIV outcomes among the broader population, FSW face intersecting stigmas that hinder access to high quality care. Our study showed that barriers to HIV services exacerbate the unmet family planning (FP) needs of FSW. Participants reported unsafe client encounters where violence and intimidation factors were used to forego condom use. Younger FSW appeared to be at greater risk of power imbalances during condom negotiation, suggesting a higher risk of HIV acquisition. Recommendations for FSW-differentiated services that address various levels of FP and HIV prevention and counselling highlight the opportunity that effective integration of services could have at addressing both the SRH and HIV prevention needs of FSW.

FSW identified condoms as the most effective method of protection against the spread of infection and HIV. A combination of modern and traditional contraceptive methods were discussed, including the use of vinegar and cotton leaves, which are not one of the known effective methods promoted by health agencies including the Côte d’Ivoire Ministry of Health, WHO and CDC [34–36]. While some studies have explore the effectiveness of traditional methods in preventing pregnancy, there is a lack of evidence that these methods are safe and effective [37, 38]. These findings are consistent with a study in Abidjan, Côte d’Ivoire that supported the integration of FP and antenatal services into HIV prevention due to low contraceptive use, particularly among young FSW, and a high burden of unintended pregnancy [39]. Inconsistent use of contraception together with the use of traditional methods highlight unmet programmatic needs for contraceptive counselling that exist beyond condom usage. This finding offers an opportunity to engage further with contraceptive options including oral contraception and implants, while also highlighting an opportunity for engagement with PrEP to protect against HIV due to the risks of exposure through condomless sex and the higher numbers of sexual partners present in sex work. Increasing dual protection against STIs/HIV and unintended pregnancy is possible through an integrated and targeted approach [40]. Previous studies have also found that integration of FP services into HIV care and treatment programming can decrease costs for service delivery, increase client knowledge and awareness, and increase usage of modern contraceptive methods [41].

While there was high knowledge of condom efficacy, this awareness did not translate directly into practice of condom use. Similar trends were found among young people in Uganda, where high knowledge of FP, STIs, and HIV in the context of structural risks did not prevent engaging in condomless sex, high numbers of sexual partners, or engaging in sex under the influence of alcohol or drugs [42]. Our findings show that the frequency of condom usage varied depending on FSW familiarity with the customer which translated into a feeling of trust and stability with the client. Additional factors that influenced the occurrence of condomless sex included sexual violence and difficulty in condom negotiation due to power asymmetry and financial pressures. Some participants also discussed experiences of condoms breaking and agreeing to continue without a condom for increased pay. In the National Strategic Plan, 62% of FSW in Côte d'Ivoire reported experiences of acceptance of higher pay for sex without a condom [43]. Age and time in sex work demonstrated age as a risk factor for HIV transmission and inconsistent condom use, particularly as younger FSW in this study were less likely to have power in condom negotiations. This finding is consistent with previous studies that report differential condom use due to coercion, economic marginalization, and physical and sexual violence [44–46]. An analysis of HIV prevalence among young FSW reported that across nine Sub-Saharan African countries, the burden of HIV was already high by the age of 18 [47]. This finding underlines the need to address the magnified risk of young women being exposed to life-threatening power dynamics and sexual exploitation or violence.

The integration of FP and HIV services in low and middle-income countries has been found to increase dual and modern contraception use [48]. An integrated approach combining routine screening for FP needs and non-condom FP method availability at FSW Drop-In Centers (DIC) in Kenya found a positive effect on condom usage [49]. Integrated services that are targeted at FSW, including family planning services and HIV counselling and testing, are needed to address the SRH needs of FSW. Many FSW pursued care in nearby towns or distant localities due to anticipated stigma. There was a consensus that FSW-differentiated services should prioritize qualities of compassion, empathy, and confidentiality among service providers. There existed an underlying mistrust of healthcare workers among FSW caused by experiences of stigma and breaches in confidentiality. The “one-stop shop” integration model provides an opportunity to decentralize HIV care and incorporate family planning services within the same clinic by the same provider. Similarly, in Tete, Mozambique, FSW reported high satisfaction with an overnight clinic offering integrated FSW-specific HIV and SRH services, with experiences of stigma and privacy breeches at public facilities [50]. Services specific to the needs of FSW can reduce the barriers to receiving care while fostering open discussions about sexual practices and behaviors. Integrated programs that prioritize a rights-affirming approach in these facilities and services are needed to minimize the stigma and coercion that further threatens FSW right to health [51]. These findings indicate the importance of strengthening integration efforts and providing FSW-differentiated care that is safe and of high quality.

Legal restrictions on abortion increase the vulnerability of FSW to unsafe and dangerous termination of pregnancy. Unintended pregnancy was discussed across all five cities, and while reasons varied, women were often left to terminate pregnancies with little to no medical or emotional support, or post-abortive care. In 2020, the 1-year incidence of abortion in Côte d’Ivoire was reported at 27.9 per 1000 women of reproductive age [52]. While FSW-specific data were not included, it can be expected that this rate may be higher due to the additional barriers to care related to sex work stigma and HIV prevalence. Specifically, FSW in this study anticipated stigma and discrimination when disclosing their sex worker status in hospitals or other public healthcare facilities. By addressing FP services and increasing contraceptive knowledge, it is possible to address the unmet need of those who prioritize pregnancy prevention while also promoting HIV prevention measures [4]. Appropriate counselling and care are needed to prevent future unintended pregnancy, highlighting a space for programmatic efforts to broaden existing HIV care services to better engage with FSW. Similar experiences of unmet need were found among FSW in Cameroon, where results showed many accounts of unintended pregnancy and termination of pregnancy coupled with low use of non-barrier contraception, as well as inconsistent condom use [53]. These findings suggest that integrated reproductive health services should include diverse FP methods including non-barrier methods of contraception and discussions of fertility goals to address the prevalence of unintended pregnancy, protect from HIV/STI transmission, and provide more care overall.

Some FSW continued to work until childbirth, while others remained at home or did not continue to work. Continuing sex work during pregnancy further exposed the individual to the vulnerabilities rooted in sex work including HIV transmission, STIs, or experiences of violence. Financial and parental obligations can shape the pregnancy experience of FSW. While some participants were able to use hospitals for childbirth, others relied on their families or other FSW mothers for delivery. Therefore, it is necessary that SRH support extends beyond the prevention of unintended pregnancy or disease transmission. The effective integration of HIV and SRH services can provide opportunities to receive ongoing testing and treatment services, while seeking antenatal care. Given ongoing acquisition risks during pregnancy, there is a need to keep testing, more than the standard of testing once during pregnancy, to avoid vertical transmission [54]. This is particularly important as the occupational risks of sex work can increase risks of STI acquisition during pregnancy [55], highlighting the need for services tailored to FSW. A systematic review of the evidence for integration SRH/HIV program implementation reinforces the efficacy of this approach across sub-Saharan Africa, highlighting the need for FP integration to address the high burden of unintended pregnancies, STIs, and HIV [56]. In the WHO guidelines for linkage between HIV services and SRH care, maternal and child health and wellbeing is listed as one of the key priorities under this integrated approach [57]. Increased research and programmatic efforts are required to explore the reproductive health needs of FSW who are mothers. By doing so, it may be possible to not only address improved SRH health outcomes, but also decreased vertical transmission of HIV.

Within this analysis, it is important to recognize the primary author’s identity as a white-passing first-generation Canadian citizen, who does not have lived experience themselves as a FSW. The data analyses occurred through ongoing conversation with the ENDA Santé team. COVID-19 travel restrictions during the study period restricted the author from working directly with the ENDA Santé team in Côte d'Ivoire. Both the primary author and the ENDA Santé team coded the transcripts and conducted thematic analysis separately, with results being compared to each other for consistency. Within the research team, there was a strong commitment to understanding the systems of privilege that could influence and instill any inherent biases and stereotypes. This awareness was at the forefront of the data analysis processes to ensure that the findings and discussions were conducted using a compassionate and culturally-safe approach.

The findings here are reported in light of several limitations. Several participants in this study had previously accessed resources or were knowledgeable about local NGOs. They were also selected from cities where there was a greater NGO presence. There are still many FSW that were not included, and future studies should strive to connect with these participants and engage with further cities across Côte d'Ivoire.

## Conclusions

Taken together, these results underline gaps in the health system for FSW and reinforce the benefits of integrating SRH programming into existing HIV services. FSW face specific barriers to care resulting in high sustained risks for both unintended pregnancy and HIV. Evidence-based implementation of targeted services is needed to ensure that as these programs move forward, FSW are not left behind in advances for both HIV, reproductive health, and the integration of the two. Strategic programming with HIV integration will work toward ensuring that FSW are empowered with the knowledge to protect themselves against HIV, unintended pregnancies, and adverse reproductive health outcomes, embodying a dignified approach to health and human rights.

## Data Availability

The datasets used and/or analysed during the current study are available from the corresponding author on reasonable request.
